# The Moderating Role of Trait Anxiety in the Relationship Between Acute Aerobic Exercise and Cognitive Control in College Students

**DOI:** 10.1155/da/1381460

**Published:** 2026-09-28

**Authors:** Zongyu Yang, Xiaoming Liu, Yi Tang, Long Yin, Fang Li

**Affiliations:** ^1^ College of Physical Education, Hunan Normal University, Changsha, China, hunnu.edu.cn; ^2^ Yali High School, Changsha, China; ^3^ College of Physical Education, Hunan First Normal University, Changsha, China, hnfnu.edu.cn

**Keywords:** acute aerobic exercise, cognitive control, college students, moderating effect, trait anxiety

## Abstract

**Objective:**

This study aimed to introduce trait anxiety as an individual‐difference factor and further examine the complex relationship between acute aerobic exercise and cognitive control in college students.

**Method:**

A mixed experimental design was adopted. Trait anxiety was assessed using the State‐Trait Anxiety Inventory (STAI) and used to classify participants into high‐ and low‐trait‐anxiety groups. Cognitive control was measured with the AX continuous performance task (AX‐CPT). The intervention conditions consisted of 20 min of seated rest and moderate‐intensity acute aerobic exercise. A 2 (intervention: acute aerobic exercise vs. rest) × 2 (time: pretest vs. posttest) × 2 (trait anxiety: high vs. low) repeated‐measures analysis of variance (ANOVA) was conducted.

**Results:**

Only participants with high trait anxiety showed a significant reduction in posttest reaction time on BX trials, an index of proactive control, following acute aerobic exercise (*p* < 0.001), with no significant change in accuracy (*p* > 0.050). In addition, the proactive behavioral index (PBI) for error rate [PBI(error)] significantly increased in this group after exercise (*p* = 0.031). No significant changes were observed in *d*′(Proactive), A‐cue bias, PBI(RT), PBI(composite), or indices of reactive control under any intervention condition. Among participants with low trait anxiety, neither intervention produced significant changes in proactive control, as reflected by BX trials, or reactive control, as reflected by AY trials and *d*′(Reactive) (all *p* *s* > 0.050).

**Conclusion:**

Trait anxiety exerted a significant influence on the relationship between acute aerobic exercise and cognitive control, with this effect being particularly evident in reaction time during proactive control. These findings suggest that future exercise interventions should incorporate individuals’ baseline cognitive control characteristics to develop more precise and individualized exercise prescriptions.

## 1. Introduction

Cognitive control, also referred to as executive control [1], is a high‐level cognitive function that regulates individual behavioral responses through both top‐down and bottom‐up attentional control processes [1, 2]. It enables individuals to inhibit irrelevant distractions and maintain goal‐directed behaviors, particularly in the face of complex tasks or conflicting situations. This function typically reaches optimal performance from adolescence through mid‐adulthood [3], rendering it especially developmentally significant for college students during this period. Moreover, cognitive control serves as a critical foundation for effectively allocating and flexibly shifting attention [4] and plays a positive role in supporting divergent thinking by suppressing irrelevant stimuli. Notably, cognitive control exerts a significant facilitating effect on prospective memory performance, thus contributing to the consolidation of academic knowledge among students. More importantly, robust cognitive control is essential for inhibiting addictive behaviors [5, 6], which has profound implications for individual social development. In light of its importance, enhancing cognitive control in college students is expected to better equip them for handling complex task demands and to lay a solid foundation for personal growth and future societal engagement.

To promote the healthy development of cognitive control, scholars have increasingly focused on the influence of everyday behavioral activities. Among these, physical exercise has garnered particular attention due to its nonpharmacological nature and low cost. Empirical research has demonstrated that chronic engagement in physical activity yields significant and lasting improvements in cognitive control [7–9]. In addition, a growing body of evidence suggests that acute physical exercise also produces positive effects on cognitive control among college students [10], as reflected in significantly enhanced task performance in terms of reaction times and/or accuracy following the intervention [11]. Some studies further highlight that moderate‐intensity acute aerobic exercise may offer the most pronounced benefits [12] and may represent the optimal intensity for intervention purposes [13]. Overall, both chronic and acute aerobic exercise have been shown to produce substantial improvements in cognitive control [14], providing valuable theoretical guidance for the positive development of college students. However, as research progresses, the consistency and stability of cognitive outcomes following acute exercise interventions remain a topic of considerable debate [15–17]. Certain studies report no significant changes in reaction times or accuracy in cognitive control tasks after acute aerobic exercise among college students [10], raising concerns about the generalizability and mechanisms underlying these effects.

To account for the inconsistent relationship between acute aerobic exercise and cognitive control in college students, previous studies have examined potential explanatory factors from two main perspectives: intervention characteristics and participant‐related characteristics. In terms of intervention characteristics, exercise intensity and duration are core components of acute aerobic exercise prescriptions as they may influence cognitive control performance by modulating physiological arousal, fatigue load, and the availability of cognitive resources [14]. Relevant evidence suggests that moderate‐intensity acute aerobic exercise lasting 10–20 min is more likely to elicit stable cognitive‐enhancing effects [14, 15, 18]. However, exercise prescription factors mainly delineate the boundary conditions of the exercise stimulus itself and remain insufficient for explaining the heterogeneity of cognitive benefits. To address this limitation, researchers have further examined participant‐related characteristics, which may represent another important source of variability in the equivocal relationship between acute aerobic exercise and cognitive control. Previous studies have shown that factors such as cardiorespiratory fitness, body mass index (BMI), and age can significantly influence cognitive control outcomes following acute aerobic exercise [9, 19]. For example, compared with younger adults, older adults appear more likely to exhibit improvements in cognitive control after acute aerobic exercise [20], highlighting the important role of individual characteristics in shaping the exercise–cognition relationship.

Taken together, previous studies have provided important evidence for explaining the inconsistent relationship between acute aerobic exercise and cognitive control from two major perspectives: exercise prescription characteristics and participant‐related characteristics. Specifically, exercise intensity and duration primarily delineate the boundary conditions under which external exercise stimuli may facilitate cognitive control, whereas participant‐related characteristics further suggest that the same acute aerobic exercise intervention may yield different cognitive benefits depending on individual differences. However, existing research has mainly focused on demographic or physiological characteristics, such as age, cardiorespiratory fitness, and BMI, while providing relatively limited explanations of how these characteristics are translated into differences in cognitive benefits. More specifically, the influence of participant‐related characteristics on the cognitive outcomes of acute aerobic exercise may be closely associated with individuals’ baseline level of cognitive control. Previous evidence indicates that individuals with relatively poorer initial cognitive control performance or lower cognitive control efficiency may be more likely to show cognitive improvements following acute aerobic exercise [21]. This suggests that stable individual characteristics that shape cognitive control functioning may provide an important entry point for understanding individual differences in the cognitive benefits of acute aerobic exercise. Based on this rationale, trait anxiety can be considered an important psychological characteristic that influences cognitive control. Eysenck’s Attentional Control Theory proposes that anxiety occupies individuals’ limited cognitive resources, thereby impairing goal‐directed processing and attentional control efficiency [22]. From the perspective of the dual mechanisms of cognitive control, proactive control, compared with reactive control, relies more heavily on the sustained active representation of goal‐relevant cues and is therefore a more resource‐demanding processing mode. Accordingly, proactive control may be particularly sensitive to anxiety‐related depletion of cognitive resources and may show more pronounced impairment [22]. In addition, compared with state anxiety, trait anxiety is more stable and enduring and may exert long‐term influences on individuals’ allocation of cognitive resources and cognitive control performance [23, 24]. Therefore, differences in cognitive control associated with varying levels of trait anxiety may further shape the cognitive benefits observed after acute aerobic exercise. Although some studies have begun to examine the relationship between acute aerobic exercise and cognitive control in individuals with different levels of trait anxiety [25], further investigation is still needed because of differences and limitations in study populations, assessment tools, and the selection of cognitive control indices. This issue is particularly relevant for college students, who are exposed to multiple sources of stress, including academic demands, employment competition, and social adaptation, making anxiety relatively common in this population. When anxiety is not effectively alleviated over time, it may be reflected in higher and relatively stable levels of trait anxiety, which may further influence cognitive control performance. Accordingly, examining the moderating role of trait anxiety in the relationship between acute aerobic exercise and cognitive control among college students may not only extend theoretical explanations of individual differences in the cognitive benefits of acute aerobic exercise but also provide a new perspective for exercise‐based interventions aimed at improving cognitive control in this population. Based on the above analysis, the present study proposed the following hypotheses: (1) compared with individuals with low trait anxiety, individuals with high trait anxiety would exhibit relatively lower proactive control efficiency, and (2) compared with individuals with low trait anxiety, individuals with high trait anxiety would be more likely to obtain cognitive control benefits from acute aerobic exercise.

## 2. Participants and Methods

### 2.1. Participants

Recruitment was conducted at a university in Nanning, Guangxi, China, from September to October 2023. The inclusion criteria were as follows: (1) full‐time undergraduate students; (2) good physical health, with no family history of hereditary diseases, no cardiovascular conditions such as hypertension or hyperglycemia, and no neurological or cognitive disorders; (3) no contraindications to physical exercise, possessing sufficient physical capability to engage in moderate‐to‐high‐intensity physical activity; and (4) trait anxiety scores ≥43.310 + 9.200 or ≤43.310 − 9.200 points [26]. Furthermore, participants were required to sign an informed consent form before officially participating in the experiment, and they were compensated with 60 RMB. The present study adopted a 2 (intervention condition: exercise intervention vs. rest intervention) × 2 (measurement time: pretest vs. posttest) × 2 (trait anxiety level: high trait anxiety vs. low trait anxiety) within‐subject repeated‐measures design. An a priori sample size estimation was conducted using G ^∗^Power, with the statistical test specified as repeated measures, within factors under *F* tests. The parameters were set as follows: effect size *f* = 0.20, significance level *α* = 0.05, statistical power 1 − *β* = 0.80, correlation among repeated measures *r* = 0.50, and no sphericity correction *ε* = 1. The results indicated that a minimum sample size of 24 participants would be required. Considering potential attrition during the experiment, the planned sample size was set above 24 participants, and participant recruitment was conducted through voluntary student sign‐ups. A total of 61 college students were initially recruited, but 3 participants withdrew due to exercise‐induced physical discomfort, 2 dropped out due to medical leave, and 4 were excluded for having low accuracy in cognitive data. Ultimately, 52 students (26 males and 26 females) completed the entire experiment. This study was approved by the ethics committee of the institution (Ethics Approval Number 202110608002).

The participant details are presented in Table 1. The results showed significant differences in anxiety scores (all *p* < 0.001), with the high trait anxiety group scoring significantly higher than the low trait anxiety group. Furthermore, the high trait anxiety group’s scores were much higher than the normative data for trait anxiety in Chinese college students (43.310 ± 9.200) [26]. No significant gender differences were found within each group (all *p* > 0.050). The results also revealed significant differences in depression scores between the trait anxiety groups (*p* < 0.001), with the high trait anxiety group showing significantly higher depression scores than the low trait anxiety group.

**Table 1:** Characteristics of participants.

Indicator	Low trait anxiety group (13 male, 13 female)	High trait anxiety group (13 male, 13 female)	*F*	*p*
Age (years)	20.077 ± 1.093	20.115 ± 1.143	0.015	0.902
Height (cm)	167.900 ± 0.063	169.300 ± 0.059	0.666	0.418
Weight (kg)	58.327 ± 12.988	58.846 ± 11.728	0.023	0.880
BMI (kg/m^2^)	20.534 ± 3.608	20.364 ± 2.870	0.035	0.852
Trait anxiety score	26.913 ± 3.837	54.565 ± 1.502	98.636	<0.001
Depression score	3.539 ± 1.140	7.154 ± 2.034	62.542	<0.001

Abbreviation: BMI, body mass index.

### 2.2. Methods

#### 2.2.1. Fitness

The intervention involved moderate‐intensity acute aerobic exercise, and certain physical diseases or impairments in participants could potentially affect their health. Therefore, the Physical Activity Readiness Questionnaire (PAR‐Q) was used to screen participants for health conditions to ensure safety during the experiment. This questionnaire was initially developed by the Canadian Society for Exercise Physiology [27] and is designed for health risk screening prior to exercise for individuals aged 15–69. It has since been continually updated and refined by subsequent scholars and is now applicable to a broader population to identify potential health risks.

#### 2.2.2. Trait Anxiety

Participants were grouped based on the State‐Trait Anxiety Inventory (STAI), Chinese version. The Chinese version was revised by scholars Li and Qian [26] at Peking University in 1995, establishing a normative dataset for trait anxiety in Chinese college students (43.310 ± 9.200). The questionnaire uses a 4‐point Likert scale with 20 items, and reverse scoring is applied to items 2, 5, 8, 9, 11, 12, 15, 17, 18, and 20. The Cronbach’s *α* reliability coefficient of the scale was 0.882. Scores ranged from a minimum of 20 to a maximum of 80. A score higher than one standard deviation (SD) above the mean of the normative sample is classified as high trait anxiety, while a score lower than one SD below the mean is classified as low trait anxiety [26].

#### 2.2.3. Depression

Previous research suggests that prolonged anxiety may also be accompanied by the onset of depressive symptoms. Therefore, to reduce measurement error in the study’s results, depression was included as a crucial control variable. Depression levels were assessed using the Beck Depression Inventory (BDI‐II, Chinese version) [28]. The BDI‐II consists of 21 items, scored using a 4‐point Likert scale, with a total score ranging from 0 to 63 (0–13 indicating no depression; 14–19 indicating mild depression; 20–28 indicating moderate depression; and 29–63 indicating severe depression) [28]. The BDI‐II has been validated through large‐sample testing in China (with a Cronbach’s *α* reliability coefficient of 0.890 and a split‐half reliability of 0.879) [28].

#### 2.2.4. Cognitive Control

The AX‐CPT (AX continuous performance task) paradigm is a classical assessment tool for cognitive control, primarily composed of cue stimuli and probe stimuli. Initially, a blue cue stimulus appears on the screen for 500 ms, followed by a 3000 ms white fixation point “+++.” Afterward, a white probe stimulus appears for 500 ms. Participants are required to prepare for a target response to “X” following the presentation of cue “A,” while all other cues are nontarget responses. The main experiment consists of 4 blocks, with each block containing a sequence of AX, AY, BX, and BY trials, totaling 40 trials per block and 160 trials in total. Among these, AY and BX reflect reactive control and proactive control, respectively (as shown in Figure 1).

**Figure 1:**
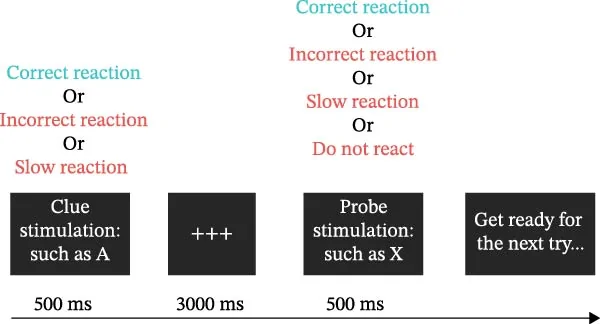
Operation process of the cognitive control task (AX‐CPT).

#### 2.2.5. Manipulation Check

To further observe the physiological changes (such as heart rate, fatigue, and emotions) and cognitive load changes before, during, and after the acute aerobic exercise intervention, the following measurement tools were used to provide more specific data feedback.

##### 2.2.5.1. Emotional Responses

The SAM scale was used to assess the emotional responses of participants in the high and low trait anxiety groups before the intervention [29]. The scale includes two dimensions: arousal and valence, with Cronbach’s *α* coefficients of 0.630 and 0.980, respectively. It consists of three main evaluation components: pleasure, arousal, and approach motivation. Pleasure is rated from 1 (very unpleasant) to 9 (very pleasant); arousal from 1 (low arousal or calm) to 9 (high arousal or excited); and approach motivation from 1 (avoidance) to 9 (approach).

##### 2.2.5.2. Cognitive Load

The NASA‐TLX scale was used to evaluate the cognitive load of the participants during the AX‐CPT task. The NASA‐TLX is widely regarded as an effective tool for assessing cognitive load and has been extensively used in academic research in China [30]. This scale includes six subdimensions: physical demand, mental demand, time demand, performance, effort, and frustration. The split‐half reliability and Cronbach’s *α* coefficient of the scale were both greater than 0.800. It is also commonly used in sports and cognitive research to assess the cognitive load during task performance.

##### 2.2.5.3. Mental Effort

The PAAS scale, developed by Paas [31], was used to evaluate the mental effort participants invested during the AX‐CPT task. The Cronbach’s *α* coefficient for this scale was 0.900. Scores range from 1 (very low effort) to 9 (very high effort), indicating the level of effort invested in the task.

##### 2.2.5.4. Exercise Experience

The RPE scale was used to assess the participants’ physical exertion during the exercise intervention [32]. The scale ranges from 6 (no exertion at all) to 20 (extremely difficult). This scale evaluates overall physical exertion rather than focusing on specific body parts, with an intraclass correlation coefficient (ICC) of 0.980. The feeling scale (FS) was used to assess emotional changes during exercise [33], with a scoring range from −5 (extremely negative emotions) to +5 (extremely positive emotions).

### 2.3. Experimental Design

The experiment consists of three stages (as shown in Figure 2): pretest of the AX‐CPT task, intervention, and posttest of the AX‐CPT task. To minimize potential carryover effects, the two experimental sessions for each participant were scheduled at least 3 days apart. Furthermore, to minimize experimental bias, a rigorous double‐blind design was implemented in this study—neither the experimenter nor the participants were informed of the specific aims of the experiment or the hypothesized relationships among variables, thereby reducing the potential influence of expectancy effects and subjective bias on the outcomes.

**Figure 2:**
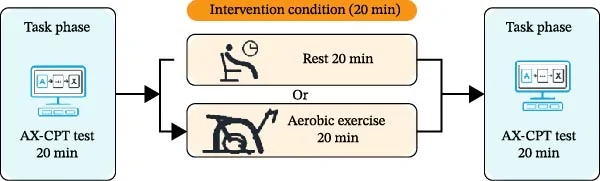
Experimental procedure.

In the initial AX‐CPT task, participants were required to learn the task procedure, followed by practice trials. The task proceeds to the formal test once participants achieve a 70% or higher accuracy rate. The AX‐CPT paradigm consists of 4 blocks, with a 1‐min rest between adjacent blocks. During the rest periods, participants were asked to complete the NASA‐TLX and PAAS scales to record the cognitive load and mental effort experienced during task performance.

To avoid measurement bias caused by intervention order effects, the study balanced the intervention sequence. Prior to the intervention, participants completed the RPE and FS scales to record their physical sensations. Before the acute aerobic exercise intervention, the participants’ resting heart rate was used to calculate the target exercise intensity. Based on the applicability of moderate‐intensity exercise and fatigue considerations, the exercise intensity was set to moderate: exercise heart rate = ([220‐age]‐resting heart rate) × (55%–65%) + resting heart rate [34]. Participants first performed a 5 min warm‐up on a stationary bicycle without resistance, with adjustments based on individual differences. After the warm‐up, the exercise intensity was gradually increased to reach the target heart rate. Once the target heart rate was achieved, the exercise was formally recorded using a Polar watch. The exercise session lasted 20 min with continuous activity, maintaining the heart rate within the moderate‐intensity zone. During the session, participants completed the RPE and FS scales at 5 min intervals to record their physical sensations. After the 20 min exercise, the Polar watch data was saved, and participants completed a 2 min cool‐down on the bike to gradually lower their heart rate and prevent injury from sudden cessation. The rest intervention followed a similar process to the exercise intervention, with participants remaining seated and refraining from using phones or engaging in conversation. The RPE and FS scales were also completed at four time points during the rest period.

The posttest AX‐CPT task was identical to the pretest, but participants did not undergo practice trials and directly proceeded to the formal task.

### 2.4. Statistic Analysis

Cognitive task data were preprocessed as follows: first, trial‐level validity screening was conducted on the raw data. Trials with reaction times shorter than 100 ms, longer than 2000 ms, or with no response were excluded as invalid trials. Second, reaction time indices were calculated based only on the correct trials. For each participant, experimental condition, and trial type, the mean and SD of reaction times for correct trials were computed, and outlying reaction times exceeding the mean ± 3 SDs were further removed. The resulting reaction time data from the correct trials were used for subsequent reaction time‐related statistical analyses.

It should be noted that incorrect trials were not included in the calculation of mean reaction times but were retained for the calculation of accuracy, error rates, and error‐rate‐based derived indices, thereby ensuring the validity of error‐rate‐related results. To further examine changes in individual cognitive control strategies, this study adopted three types of indices: the discriminability index d‐context, the proactive behavioral index (PBI), and A‐cue bias. The specific formulas are presented in Table S1.

Specifically, *d*(Proactive) was used to reflect the implementation level of proactive control, with larger values indicating a greater ability to use cue information to maintain task goals. *d*(Reactive) was used to reflect the implementation level of reactive control, with larger values indicating a stronger ability to adjust responses after the presentation of the probe stimulus. PBI was calculated separately based on error rates [PBI(error)] and reaction times [PBI(RT)]. Values greater than 0 indicate a stronger tendency to adopt a proactive control strategy, whereas values below 0 indicate a stronger tendency to adopt a reactive control strategy. In addition, a composite PBI was calculated by standardizing and averaging the error‐rate‐based and reaction‐time‐based PBI scores, thereby providing an integrated measure of proactive control implementation. The A‐cue bias was used to reflect the tendency to prepare a target response based on the A cue; changes in this index further helped characterize individuals’ use of cue information and strategic adjustment. Taken together, these indices enabled a more detailed examination of changes in cognitive control strategies following acute aerobic exercise from multiple perspectives, including reaction time, error rate, and derived control indices.

A 2 (intervention: acute aerobic exercise, rest) × 2 (time: pretest, posttest) × 2 (trait anxiety: high trait anxiety, low trait anxiety) repeated‐measures analysis of variance (ANOVA) was conducted to examine the influence of trait anxiety on the relationship between acute aerobic exercise and cognitive control. This study primarily focused on exploring the moderating effect of trait anxiety, with particular attention to the intervention × time × trait anxiety interaction effect. If the interaction effect was significant, simple effect analysis was performed to clarify the specific changes in cognitive control following acute aerobic exercise in the high‐ and low‐trait anxiety groups. In the case of significant main effects, Bonferroni corrections were applied for multiple comparisons. The results are presented as mean ± SD (M ± SD), with statistical significance set at *p* < 0.050.

## 3. Results

### 3.1. Manipulation Check of Acute Aerobic Exercise and Rest Intervention

A 2 (intervention: acute aerobic exercise, rest) × 2 (time: pretest, posttest) repeated measures ANOVA revealed significant between‐group differences in heart rate, subjective exertion, mental effort, and subjective fatigue (*p* *s* < 0.050) as shown in Table S2. Specifically, the intervention × time interaction effect for heart rate was significant [*F*(1, 51) = 1197.443, *p* < 0.001, $\eta_{p}^{2}=0.959$]. Simple effects analysis showed that the mean heart rate during acute aerobic exercise (149.954 ± 2.688) was significantly higher than during the rest intervention (82.269 ± 1.286). These results collectively suggest that acute aerobic exercise met the predefined moderate‐intensity criteria for this study, and the rest intervention was effectively implemented as expected.

### 3.2. Manipulation Check of AX‐CPT Task Execution in High and Low Trait Anxiety Groups

A 2 (intervention: acute aerobic exercise, rest) × 2 (time: pretest, posttest) × 2 (trait anxiety: high trait anxiety, low trait anxiety) repeated measures ANOVA revealed no significant main effects or interaction effects for overall cognitive load (*p* *s* > 0.050). However, in the analysis of dimensions such as psychological effort and mental demand, it was found that the main effect or interaction effect results were significant (*p* *s* < 0.050). Comprehensive evidence indicates that acute aerobic exercise significantly improved the utilization of cognitive resources and enhanced the executive efficiency of cognitive processes in both groups of individuals with trait anxiety. Especially in the dimension of psychological effort, it was found that individuals in the high‐trait anxiety group made lower efforts, indicating that they achieved more significant benefits. These findings are summarized in Table S3.

### 3.3. The Relationship Between Acute Aerobic Exercise and Active Control in High and Low Trait Anxiety Groups

#### 3.3.1. Proactive Control (BX) Response Time

The repeated measures ANOVA revealed that the main effect of intervention, the time × trait anxiety interaction, and all other effects were not significant (*p* *s* > 0.050), with the sole significant effect being the time × intervention × trait anxiety three‐way interaction, *F*(1, 46) = 5.223, *p* = 0.027, $\eta_{p}^{2}=0.102$.

Simple effects analysis of this interaction (see Figure 3) indicated that, exclusively under the acute aerobic exercise intervention, the high trait anxiety group exhibited a significant reduction in BX trial reaction times from pretest to posttest, *F*(1, 46) = 10.221, *p* = 0.003, $\eta_{p}^{2}=0.182$. Posttest reaction times (318.308 ± 69.407) were significantly lower than pretest reaction times (375.577 ± 74.952). In contrast, no significant pre‐to‐post changes in BX reaction time were observed in the low trait anxiety group under either the acute aerobic exercise or the rest intervention (*p* *s* > 0.050), as illustrated in Figure 3. Under the rest intervention, no significant between‐group or within‐group differences in BX reaction time emerged between the high and low trait anxiety groups (*p* *s* > 0.050). Further simple effects analysis demonstrated that, relative to the rest intervention, acute aerobic exercise exerted a more pronounced facilitatory effect on BX reaction time in the high trait anxiety group, *F*(1,46) = 10.702, *p* = 0.002, $\eta_{p}^{2}=0.185$. Taken together, these results indicate that acute aerobic exercise selectively shortened proactive control reaction times in individuals with high trait anxiety but did not affect those with low trait anxiety.

**Figure 3:**
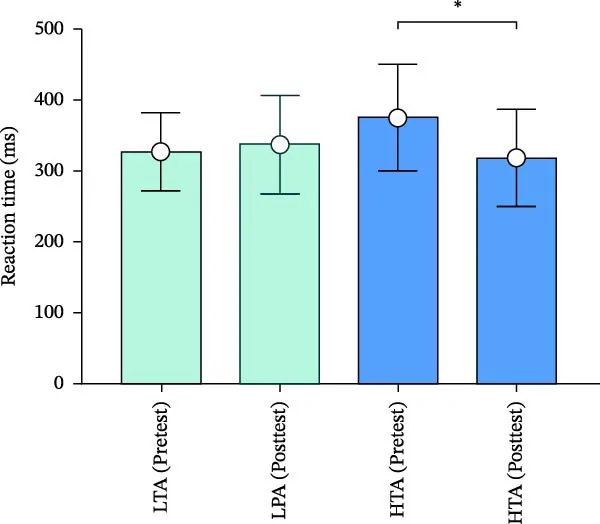
Changes in the response time of each BX test in each group under exercise intervention.  ^∗^ indicates a significant difference within the group between pre‐ and post‐measurements, *p* < 0.05.

#### 3.3.2. The Accuracy Rate of Proactive Control (BX)

The repeated measure ANOVA results for BX accuracy are presented in Table S4. Neither the main effects nor any interaction effects reached significance (*p* *s* > 0.050), indicating that proactive control accuracy remained stable across conditions and was not modulated by the intervention.

#### 3.3.3. Other Indicators of Proactive Control

Beyond BX reaction time and accuracy, repeated measures ANOVAs on the remaining proactive control indices—including *d*′ (proactive), A‐Cue bias, PBI (RT), and PBI (composite)—yielded no significant main or interaction effects (*p* *s* > 0.050). A significant intervention × time × trait anxiety interaction was found only for the PBI (error) index, *F*(1, 46) = 4.610, *p* = 0.019, $\eta_{p}^{2}=0.114$. Simple effects analysis revealed that, under the acute aerobic exercise intervention, the high trait anxiety group showed a significant increase in PBI (error) from pretest to posttest, *F*(1, 46) = 4.934, *p* = 0.031, $\eta_{p}^{2}=0.097$. Further simple effects analysis indicated that, compared to the rest intervention, moderate‐intensity acute aerobic exercise produced a greater improvement in the PBI (error) index for the high trait anxiety group, *F*(1,46) = 10.352, *p* = 0.002, $\eta_{p}^{2}=0.184$. These results suggest that acute aerobic exercise enhanced a specific facet of proactive control—as indexed by PBI (error)—in the high trait anxiety group, while the majority of proactive control indices remained unaffected. Detailed statistics are provided in Table 2.

**Table 2:** Mean PBI (error) indicators of different trait anxiety groups.

Indicator	Assessment time point	Low trait anxiety	High trait anxiety
Rest‐PBI (error)	Pre‐test	−0.094 ± 0.427	−0.130 ± 0.455
	Post‐test	−0.290 ± 0.455	−0.422 ± 0.396
Exe‐PBI (error)	Pre‐test	−0.091 ± 0.431	−0.263 ± 0.391
	Post‐test	−0.159 ± 0.393	−0.096 ± 0.436

*Note:* Covariates: gender, age, depression score.

Abbreviations: BMI, body mass index; PBI, proactive behavioral index.

### 3.4. The Relationship Between Acute Aerobic Exercise and Reactive Control in High and Low Trait Anxiety Groups

Detailed results are presented in Tables S5–S7. No significant changes were observed in either the accuracy or reaction time of reactive control across conditions (all *p* *s* > 0.050), indicating that neither acute aerobic exercise nor rest produced detectable effects on reactive control in either the high or low trait anxiety group.

## 4. Discussion

Previous investigations into the relationship between acute aerobic exercise and cognitive control have yielded inconsistent results, suggesting that the relationship between acute aerobic exercise and cognitive control is complex and is not yet fully clarified. Therefore, this study continued to explore the relationship between acute aerobic exercise and cognitive control, with a particular focus on trait anxiety as a participant characteristic. The results indicated that acute aerobic exercise can significantly promote the positive development of individual cognitive control. Specifically, under acute aerobic exercise, participants in the high trait anxiety group exhibited significant changes in response times during the proactive control task, with posttest response times significantly lower than pretest values, indicating a significant improvement in proactive control ability in this group. However, no significant changes in cognitive control were observed in the low trait anxiety group following acute aerobic exercise, providing overall support for the moderating effect of trait anxiety.

### 4.1. The Positive Impact of Acute Aerobic Exercise on Cognitive Control

Although acute aerobic exercise did not exert a significant effect on reactive control, 20 min of moderate‐intensity acute aerobic exercise significantly shortened response times in proactive control. Specifically, the high trait anxiety group demonstrated a marked reduction in proactive control response times following the intervention, whereas no comparable change was observed in the low trait anxiety group. These results indicate that acute aerobic exercise selectively enhanced proactive control in individuals with elevated trait anxiety, providing further evidence for a positive association between acute aerobic exercise and cognitive control and underscoring the beneficial role of exercise in this domain.

One plausible mechanism underlying this effect concerns shifts in cognitive resource allocation among individuals with high trait anxiety. Prior work has shown that acute aerobic exercise, particularly endurance‐oriented training, can ameliorate negative affect in college students [35], which in turn constitutes an important pathway for augmenting positive emotional experience. Given that emotion exerts a substantial influence on cognitive control execution [36] and that higher cognitive activation facilitates more efficient conflict processing [37], individuals with high trait anxiety—who are prone to negative emotional states—may have a portion of their cognitive resources diverted by task‐irrelevant information, thereby reducing the overall efficiency of the cognitive system. Following the acute aerobic exercise intervention, participants in the high trait anxiety group reported more positive emotional scores, suggesting that the intervention may have liberated cognitive resources previously occupied by negative affect, thereby benefiting task performance during the posttest AX‐CPT.

The TLX and PASS scale data from the AX‐CPT task further corroborate this interpretation. The high trait anxiety group exhibited significant differences in cognitive load and mental effort before and after the intervention: posttest cognitive load, cognitive demand, and mental effort were all significantly lower than pretest values. These results indicate that acute aerobic exercise reduced the cognitive burden on brain regions supporting task execution in the high trait anxiety group and rendered attentional resource allocation more efficient.

Although the present study did not employ neuroimaging or neurophysiological measures, prior neuroscientific findings may illuminate the mechanisms through which exercise‐induced cognitive improvements occur. Research suggests that the most pronounced cognitive benefits of acute aerobic exercise are associated with enhanced brain activation and cerebral blood circulation [38, 39]. With respect to brain activation, increased activation broadens the functional collaboration pathways among brain regions. For example, the amygdala—a core emotional center [40]—typically exhibits excessive activation under conditions of high trait anxiety or stress [41]. Such hyperactivation renders individuals more susceptible to anxiety‐driven thoughts and negative affect [41], which in turn can impede the execution of proactive control. Acute aerobic exercise has been shown to suppress amygdala activity and reduce sensitivity to negative information [42, 43], thereby attenuating anxiety‐related interference during task performance [44]. With respect to cerebral circulation, the marked increase in cerebral blood flow promotes the transmission and exchange of neurotransmitters [45, 46]. One notable example is brain‐derived neurotrophic factor (BDNF), whose circulating levels are significantly correlated with cognitive control [47] and are acutely elevated during aerobic exercise [47]. The 20 min bout of acute aerobic exercise employed in the present study may have raised BDNF levels, thereby strengthening the connectivity of neural networks involved in broader cognitive processes [47],—particularly those subserving memory enhancement—and helping individuals sustain goal‐directed representations over extended intervals in continuous performance tasks.

Notably, despite the improvement in response time for proactive control strategies, no significant change in accuracy was observed. One interpretation concerns the characteristics of individuals with trait anxiety. In the pretest, although the high trait anxiety group did not differ significantly from the low trait anxiety group in accuracy, this level of performance appeared to be achieved at the cost of prolonged response times. Moreover, given that the mean age of participants was ~20 years—a period characterized by peak cognitive development [3]—individuals with low trait anxiety may have already approached a ceiling in task performance before the accumulation of substantial trait anxiety‐related deficits, thereby maintaining consistently high levels of accuracy. Considering that proactive control accuracy did not differ between high and low trait anxiety groups at baseline, we hypothesize that individuals with high trait anxiety may likewise have reached a ceiling in proactive control accuracy [48]. Consequently, even in the presence of a positive intervention, such as acute aerobic exercise, no significant accuracy gains were detected.

### 4.2. The Differences in the Relationship Between Acute Aerobic Exercise and Cognitive Control Between High and Low Trait Anxiety Groups

The present findings indicate that acute aerobic exercise significantly promotes proactive control performance; however, this facilitatory effect was confined to the high trait anxiety group, with no significant changes in either proactive or reactive control observed in the low trait anxiety group. These results suggest that the benefits of acute aerobic exercise for cognitive control may be selective and that trait anxiety level significantly moderates the relationship between acute aerobic exercise and cognitive control. Specifically, the magnitude of the exercise‐induced cognitive benefit appeared to increase with the severity of trait anxiety. Examining third‐variable moderating mechanisms—of which trait anxiety is one—is therefore essential for a complete understanding of the exercise–cognition relationship. Prior research has found that individuals with attention‐deficit hyperactivity disorder (ADHD) derive greater benefit from acute aerobic exercise interventions than healthy individuals [49, 50], accompanied by more pronounced frontal lobe activation [49]. Related findings similarly indicate that populations whose cognitive performance is compromised—whether by clinical conditions or by other factors—tend to benefit more substantially from acute aerobic exercise [51]. These patterns converge on a key insight: individuals with lower baseline cognitive performance may experience more pronounced improvements in cognitive control following exercise [52, 53]. Building on this observation, the concept of baseline cognitive level has garnered increasing attention as it reflects an individual’s current cognitive performance capacity [54]. Evidence suggests that individuals with lower baseline cognitive levels gain greater benefits from acute aerobic exercise [21] and that once baseline cognitive level is controlled for, acute aerobic exercise interventions yield broadly positive effects. This further implies that the baseline cognitive level may serve as an important moderator of the exercise–cognition relationship and may partly account for the inconsistencies observed in this literature. Meta‐analytic evidence similarly indicates that, relative to other age groups, prepubertal children and older adults derive greater cognitive benefits from acute aerobic exercise [52, 55]. We speculate that this pattern arises because these two populations occupy the opposing ends of the cognitive developmental trajectory: children are still undergoing cognitive maturation, while older adults are experiencing cognitive decline, and both groups, on average, have lower baseline cognitive levels than those in early adulthood [3]. They may, therefore, be more sensitive to the cognitive effects of acute aerobic exercise.

In the present study, individuals with high trait anxiety may have experienced persistent negative affect that occupied a portion of their cognitive resources, preventing their efficient deployment and leading to suboptimal proactive control performance. By contrast, the low trait anxiety group—being less susceptible to anxiety—likely possessed a higher baseline level of cognitive control. Although no significant behavioral changes were observed in the low trait anxiety group following exercise, the self‐report measures revealed a significant reduction in cognitive demand and mental effort during the posttest AX‐CPT task, indicating that, even in this group, the exercise intervention enhanced task execution efficiency and reduced the cognitive cost of task performance.

## 5. Limitations

This study examined the moderating role of trait anxiety in the relationship between acute aerobic exercise and cognitive control exclusively from a behavioral perspective. Consequently, the observed findings and their interpretations remain relatively limited. Future research should integrate neurophysiological techniques—such as electroencephalography (EEG), functional near‐infrared spectroscopy (fNIRS), and functional magnetic resonance imaging (fMRI)—to capture more detailed neural changes associated with the intervention. For instance, although individuals in the low trait anxiety group demonstrated no significant behavioral differences—likely due to a “ceiling effect”—their cognitive cost during task execution significantly decreased, suggesting that substantial neural changes may have occurred at a deeper level. Additionally, scholars in related fields have noted that although high trait anxiety exerts detrimental effects on cognitive control, a significant interaction also exists between trait anxiety and state anxiety [56, 57]. These studies suggest that among individuals high in trait anxiety, increasing levels of state anxiety may exert stronger effects on various aspects of functioning [56, 57]. Whether such an interaction also modulates the relationship between acute aerobic exercise and cognitive control remains an open question for future investigation.

## 6. Conclusion

Twenty minutes of acute aerobic exercise can bring significant positive effects to the cognitive control of college students, especially for active control. In addition, trait anxiety is an important regulatory factor in the relationship between acute aerobic exercise and cognitive control. That is, individuals with high trait anxiety are more likely to achieve the improvement benefits of acute aerobic exercise compared to those with low trait anxiety.

## Author Contributions

**Zongyu Yang**: conceptualization, methodology, formal analysis, resources, investigation, data curation, writing – original draft. **Xiaoming Liu**: validation, writing – review and editing, supervision. **Yi Tang**: conceptualization, investigation, resources. **Long Yin and Fang Li**: validation, writing – review and editing.

## Funding

This study was not funded by any individual or institution in any form throughout the entire process.

## Conflicts of Interest

The authors declare no conflicts of interest.

## Supporting Information

Additional supporting information can be found online in the Supporting Information section.

## Supporting information


**Supporting Information** Due to space constraints and to emphasize the primary research findings, data regarding manipulation checks and reactive control are provided as supplementary materials. Specifically, Table S1 details the calculation formulas for the cognitive control measurement indices. Tables S2 and S3 present the manipulation check data, reflecting detailed individual changes during the intervention and cognitive control assessment processes. Furthermore, Tables S4–S7 provide the results of repeated measures analysis of variance (RM‐ANOVA) for partial proactive control and all reactive control. For further details, please refer to the Supporting Information.

## Data Availability

The data that support the findings of this study are available from the corresponding author upon reasonable request.
