# Supplementary material for: The Moderating Role of Trait Anxiety in the Relationship Between Acute Aerobic Exercise and Cognitive Control in College Students
Source: Depress Anxiety. 2026 Sep 28;2026:1381460. doi: 10.1155/da/1381460 (PMC13617466; doi:10.1155/da/1381460)
Supplement: Supplementary file 1 [file DA-2026-1381460-s001.docx]

In the AX-CPT paradigm, a higher Discriminability Index (d-Proactive) value reflects better execution of proactive control strategies. This value can be transformed into d (Reactive), which reflects the execution of reactive control strategies, with higher values indicating better performance in reactive control. Furthermore, PBI can be calculated based on error rate (PBI-error) and reaction time (PBI-RT), both of which reflect the execution of proactive control strategies. A PBI value greater than 0 suggests a tendency to use proactive control strategies, while values less than 0 indicate a preference for reactive control strategies. Expanding on this indicator, a composite PBI (average of error rate and reaction time, both standardized) was used to reflect the execution of proactive control strategies.

Finally, A-Cue bias assesses an individual’s ability to adjust based on cue information A. The formula reflects the change in strategy execution, with higher values indicating stronger performance in reactive control strategies, and lower values suggesting weaker execution of reactive control. By integrating these additional metrics, the study provides more specific evidence supporting the changes and development of an individual's cognitive control strategies.

Supplementary Table 1 Calculation Formulas for Cognitive Control-related Indicators

| Sequence number | Formula ^[1]^ |
| --- | --- |
| 1 | Formula 1: d_（Proactive）_=[Z(AX-Correct)-Z(BX-Error)] |
| 2 | Formula 2:d_（Reactive）_=[Z(AX-Correct)-Z(AY-Error)] |
| 3 | Formula 3:PBI=(AY-BX)/(AY+BX) |
| 4 | Formula 4:$PBI(composite)=\frac{Z[PBI\left( error \right)]+Z[PBI\left( RT \right)]}{2}$ |
| 5 | Formula 5: 1/2*(Z[H]+Z[F]) |

Note: Here, Z represents the standardized score

Supplementary Table 2 Verification results during the implementation of the two interventions

|  |  | Acute aerobic exercise | | Rest | |
| --- | --- | --- | --- | --- | --- |
|  |  | Pre-test | Post-test | Pre-test | Post-test |
|  | Heart rate | 85.327±12.687 | 149.954±2.688 | 84.231±1.421 | 82.269±1.286 |
|  | Subjective exertion | 9.712±1.764 | 13.550±0.314 | 9.885±1.947 | 9.792±0.196 |
|  | Perceived exercise | 2.175±1.269 | 2.735±1.010 | 2.596±1.125 | 2.115±0.148 |
| SAM scale | Perceived pleasure | 5.115±1.530 | 6.677±0.857 | 5.827±1.043 | 5.727±0.149 |
|  | Activation | 5.231±1.182 | 5.735±0.155 | 4.923±1.412 | 4.796±0.148 |
|  | Approaching | 5.635±1.609 | 5.173±0.221 | 6.096±1.361 | 5.758±0.160 |
|  | Mental effort | 5.423±1.696 | 5.754±0.231 | 5.092±1.881 | 4.781±0.226 |
|  | Subjective fatigue | 3.596±1.796 | 5.792±0.220 | 3.308±1.651 | 3.385±0.176 |

Covariates: gender, age, BMI: Body Mass Index, depression score

Supplementary Table 3 Cognitive load and subjective psychological effort of AX-CPT tasks before and after acute aerobic exercise

|  |  | Low Trait Anxiety-Pre-test | Low Trait Anxiety - Post-test | High Trait Anxiety - Pre-test | High Trait Anxiety - Post-test |
| --- | --- | --- | --- | --- | --- |
| TLX scale | Mental demand | 11.526±8.478 | 12.244±8.223 | 15.039±11.074 | 8.846±8.427 |
|  | Physical demand | 2.269±4.962 | 2.962±5.089 | 1.615±2.526 | 2.244±2.545 |
|  | Time demand | 12.320±8.219 | 11.128±7.707 | 15.064±9.028 | 13.1667±8.168 |
|  | Effort level | 12.859±8.604 | 12.013±8.066 | 14.269±8.204 | 9.628±6.050 |
|  | Task performance | 6.718±6.377 | 5.846±6.139 | 4.667±6.629 | 3.756±4.456 |
|  | Frustration | 2.846±5.330 | 2.372±4.833 | 1.539±2.511 | 2.462±3.750 |
|  | Total cognitive load | 48.539±18.678 | 44.526±21.101 | 52.192±21.783 | 40.103±17.696 |
| PASS scale | Mental effort | 5.125±1.616 | 4.760±1.595 | 5.923±1.257 | 3.587±0.843 |

Note: SAM=Self-assessment manikin (Emotion Scale); TLX= Task load index (Cognitive Load Scale) PASS=Mental effort scale;

Supplementary Table 4 Repeated measures analysis of Variance for the accuracy rate of BX

|  | df | Mean square | F | P | $\eta_{P}^{2}$ |
| --- | --- | --- | --- | --- | --- |
| Intervention | 1 | 0.020 | 0.582 | 0.449 | 0.012 |
| Intervention×Trait anxiety | 1 | 0.006 | 0.177 | 0.676 | 0.004 |
| Time | 1 | 0.044 | 1.409 | 0.241 | 0.029 |
| Time×Trait anxiety | 1 | 0.001 | 0.003 | 0.957 | 0.000 |
| Intervention×Time | 1 | 0.000 | 0.015 | 0.905 | 0.000 |
| Intervention×Time×Trait anxiety | 1 | 0.009 | 0.322 | 0.573 | 0.007 |

Covariates: gender, age, BMI=Body Mass Index, depression score

Supplementary Table 5 Repeated measures analysis of Variance for each AY reaction time

|  | df | Mean square | F | P | $\eta_{P}^{2}$ |
| --- | --- | --- | --- | --- | --- |
| Intervention | 1 | 4067.478 | 0.866 | 0.357 | 0.018 |
| Intervention×Trait anxiety | 1 | 1999.861 | 0.426 | 0.517 | 0.009 |
| Time | 1 | 21.084 | 0.009 | 0.923 | 0.000 |
| Time×Trait anxiety | 1 | 879.711 | 0.392 | 0.534 | 0.008 |
| Intervention×Time | 1 | 909.232 | 0.312 | 0.579 | 0.007 |
| Intervention×Time×Trait anxiety | 1 | 7647.545 | 2.621 | 0.112 | 0.053 |

Covariates: gender, age, BMI=Body Mass Index, depression score

Supplementary Table 6 Repeated measures analysis of variance for the accuracy rate of AY

|  | df | Mean square | F | P | $\eta_{P}^{2}$ |
| --- | --- | --- | --- | --- | --- |
| Intervention | 1 | 0.002 | 0.136 | 0.714 | 0.003 |
| Intervention×Trait anxiety | 1 | 0.050 | 2.818 | 0.100 | 0.058 |
| Time | 1 | 0.012 | 0.910 | 0.345 | 0.019 |
| Time×Trait anxiety | 1 | 0.009 | 0.689 | 0.411 | 0.015 |
| Intervention×Time | 1 | 0.005 | 0.606 | 0.440 | 0.013 |
| Intervention×Time×Trait anxiety | 1 | 0.035 | 3.822 | 0.057 | 0.077 |

Covariates: gender, age, BMI=Body Mass Index、depression score

Supplementary Table 7 Repeated measures analysis of variance for the reactive control on other indicators (d-context)

|  | df | Mean square | F | P | $\eta_{P}^{2}$ |
| --- | --- | --- | --- | --- | --- |
| Intervention | 1 | 0.014 | 0.010 | 0.921 | 0.000 |
| Intervention×Trait anxiety | 1 | 5.315 | 3.790 | 0.058 | 0.076 |
| Time | 1 | 0.981 | 1.019 | 0.318 | 0.022 |
| Time×Trait anxiety | 1 | 0.036 | 0.037 | 0.848 | 0.001 |
| Intervention×Time | 1 | 0.121 | 0.136 | 0.714 | 0.003 |
| Intervention×Time×Trait anxiety | 1 | 2.166 | 2.427 | 0.126 | 0.050 |

Covariates: gender, age, BMI=Body Mass Index、depression score

[1]GONTHIER C, MACNAMARA B N, CHOW M,et al. Inducing proactive control shifts in the ax-cpt [J]. Frontiers in Psychology, 2016, 7: 1822.
